# Supplementary material for: Chromothripsis during telomere crisis is independent of NHEJ, and consistent with a replicative origin
Source: Genome Res. 2019 May;29(5):737–49. doi: 10.1101/gr.240705.118 (PMC6499312; doi:10.1101/gr.240705.118)
Supplement: Supplemental Material [file supp_gr.240705.118_Supplemental_file_1.zip › contigs/annotated_contigs/DB51/contig.2.DB51_length_715_mean_cov_7.44055944056.docx]

**DB51_length_715_mean_cov_7.44055944056**

```
ATATGAAGGAAAAACAATTGTAGACTCCTAAAATCAACAGTGTAATTTTTAAAATTCTGAGCAAAGGAATATTTATATCAGTTACTATT
 >chr6:164773575-164773980 + E=7e-232 p=4e-02
TTGGAGAGACCATGAATAGAGGCTAATATCAGGAGCCTTTGGTATTATTTATGCACAAATCTTAGCTTTTCTTTGTTTTTGTTGTTGCT

CACTATCATTACCAAGGGTTTTTTTTGTTTGGTTTGTTTTTTTACTTTTATTTTAGGTTCAGAGGGTCACTTGCAGGTTTGTTACATGG

GTAAATTTCATGTTGCTGAGGTTTGGTGTACAGATGATCCCATCATCCAGTAGTGAGCATAATACCCATAATACCTGATAGGTAGTTTT

TCAGCCTACACCCCTATCCCGCCTTCATCCCTCTAGTAGTCCCTAGTG|T|ATATATATAGTGTATATATATATATATACACATACATA
                                                 >chr6:164744876-164745184 - E=4e-162
TGCATATGTATTTCAAAACATCATAATGTAAACCTTAAATGTATATAAAAATAACTAATTAAAAAGAAGACACTGGAAAAATATCCTCA

ATAAAAAACCCCACAACTCACAGCAAATATTTGAAAATACGACCAAATTTTAAAATAAAATGTGAAATATTTAGCCAGGTACAGTGGCT

CACACCTATAATCCCAGCATTTTGGGAGGCCAAAGCAGGAAGATTGCTTGAGACAAGGAATTCGAGACTGGTCTGGACAACATTACAAG

ACCCT
```
